# Assessing and managing the risk of *Aedes* mosquito introductions via the global maritime trade network

**DOI:** 10.1371/journal.pntd.0012110

**Published:** 2024-04-10

**Authors:** Janna R. Willoughby, Benjamin A. McKenzie, Jordan Ahn, Todd D. Steury, Christopher A. Lepzcyk, Sarah Zohdy

**Affiliations:** 1 College of Forestry, Wildlife, and Environment, Auburn University, Auburn, Alabama, United States of America; 2 Geospatial Research, Analysis, and Services Program, Centers for Disease Control and Prevention, Agency for Toxic Substances and Disease Registry, Atlanta, Georgia, United States of America; 3 Division of Parasitic Diseases and Malaria, Centers for Disease Control and Prevention, Atlanta, Georgia, United States of America; 4 College of Veterinary Medicine, Auburn University, Auburn, Alabama, United States of America; Centers for Disease Control and Prevention, Puerto Rico, UNITED STATES

## Abstract

The global shipping network (GSN) has been suggested as a pathway for the establishment and reintroduction of *Aedes aegypti* and *Aedes albopictus* primarily via the tire trade. We used historical maritime movement data in combination with an agent-based model to understand invasion risk in the United States Gulf Coast and how the risk of these invasions could be reduced. We found a strong correlation between the total number of cargo ship arrivals at each port and likelihood of arrival by both *Ae*. *aegypti* and *Ae*. *albopictus*. Additionally, in 2012, 99.2% of the arrivals into target ports had most recently visited ports likely occupied by both *Ae*. *aegypti* and *Ae*. *albopictus*, increasing risk of *Aedes* invasion. Our model results indicated that detection and removal of mosquitoes from containers when they are unloaded effectively reduced the probability of mosquito populations establishment even when the connectivity of ports increased. To reduce the risk of invasion and reintroduction of *Ae*. *aegypti* and *Ae*. *albopictus*, surveillance and control efforts should be employed when containers leave high risk locations and when they arrive in ports at high risk of establishment.

## Author summary

Our study investigates how the global shipping network inadvertently serves as a highway for the spread of two mosquito species, *Aedes aegypti* and *Aedes albopictus*, both of which are vectors for pathogens causing diseases like dengue and Zika. We analyzed maritime movement data in the United States Gulf Coast and found that the more cargo ships that dock at a port, the higher the chance these mosquitoes will be introduced; nearly all cargo ships arriving at these ports had last visited regions where these mosquitoes thrive, significantly raising the risk of spread of these mosquitoes. Our research also suggested that proactive detection and elimination of mosquitoes from shipping containers can dramatically lower the risk of these pests establishing new colonies, even as global shipping traffic grows. Our work suggests that by enhancing surveillance and control measures at strategic points in the shipping process, we can safeguard our communities from additional mosquito introductions.

## Introduction

The globalization of trade and travel has allowed many invasive species to disperse and establish themselves in novel locations and at distances much farther than their natural dispersal abilities should allow [1,2]. These dispersal events are fueled by our increasingly interconnected world [1]. The global shipping network, which currently accounts for >80% of international trade, has expanded dramatically in the past 50 years and is expected to increase by at least 240% by 2050 [2,3]. Notably, the global shipping network acts as a significant pathway for the long-distance transport of organisms to novel locations [4,5]; aquatic species are often transported in the ballast water or attached to the hulls of vessels [6,7] whereas terrestrial species are often accidentally transported with the cargo [2,8].

International maritime trade and the global shipping network have been instrumental in the global introduction of several medically important *Aedes* spp. Mosquitoes. Most notably this includes *Aedes* (Stegomyia) *aegypti* (L.) and *Aedes* (Stegomyia) *albopictus* (Skuse) [9–11] that are the primary vectors of globally significant arboviruses including the dengue fever, chikungunya, Zika, and yellow fever viruses [12–15]. Although both species exhibit relatively short flight ranges that constrain their natural dispersal capabilities [16], the combination of their unique adaptations and long-range global shipping network enables their spread to distant locations, resulting in near-global distributions [17,18]. For example, the expansion of the *Ae*. *aegypti* range lead to a shift in blood-feeding behavior [19]: the occurrence of anthropophagy in *Ae*. *aegypti* is believed to have increased in frequency during long ship crossings in the pre-industrial era, where selection against zoophagy would have removed individuals and lineages that relied on animal meals due to a lack of availability [10]. In addition, *Ae*. *aegypti* and *Ae*. *albopictus* can oviposit in artificial containers allowing them to thrive in highly urban environments [20,21]. Adaptations to anthropogenic environments, combined with the unique ability of their eggs to survive desiccation for extended periods [20,22], have allowed these mosquitoes to be transported globally through the global shipping network via movement of potted plants and used tires [17,23,24].

While climate change alters global patterns of habitat suitability for both *Ae*. *aegypti* and *Ae*. *albopictus* [25,26], both species continue to expand in most parts of the world via new maritime introduction events and overland spread [19]. Because of widespread reductions in vector control efforts towards the end of the 20^th^ century and continuous reintroductions, *Ae*. *aegypti* has also reestablished itself in parts of its range from which it was once extirpated, including parts of the southeastern US [27,28]. The most effective strategy in limiting the spread of pest species, including *Ae*. *aegypti* and *Ae*. *albopictus*, is the implementation of effective biosecurity measures at points of entry [29] using early detection and rapid response to prevent incursion and establishment [30]. Because resources available for effective early detection and rapid response networks are generally limited, the identification of high risk locations for the importation and reintroduction of invasive species is critical for effective biosecurity [8,29,30].

**Fig 1:**
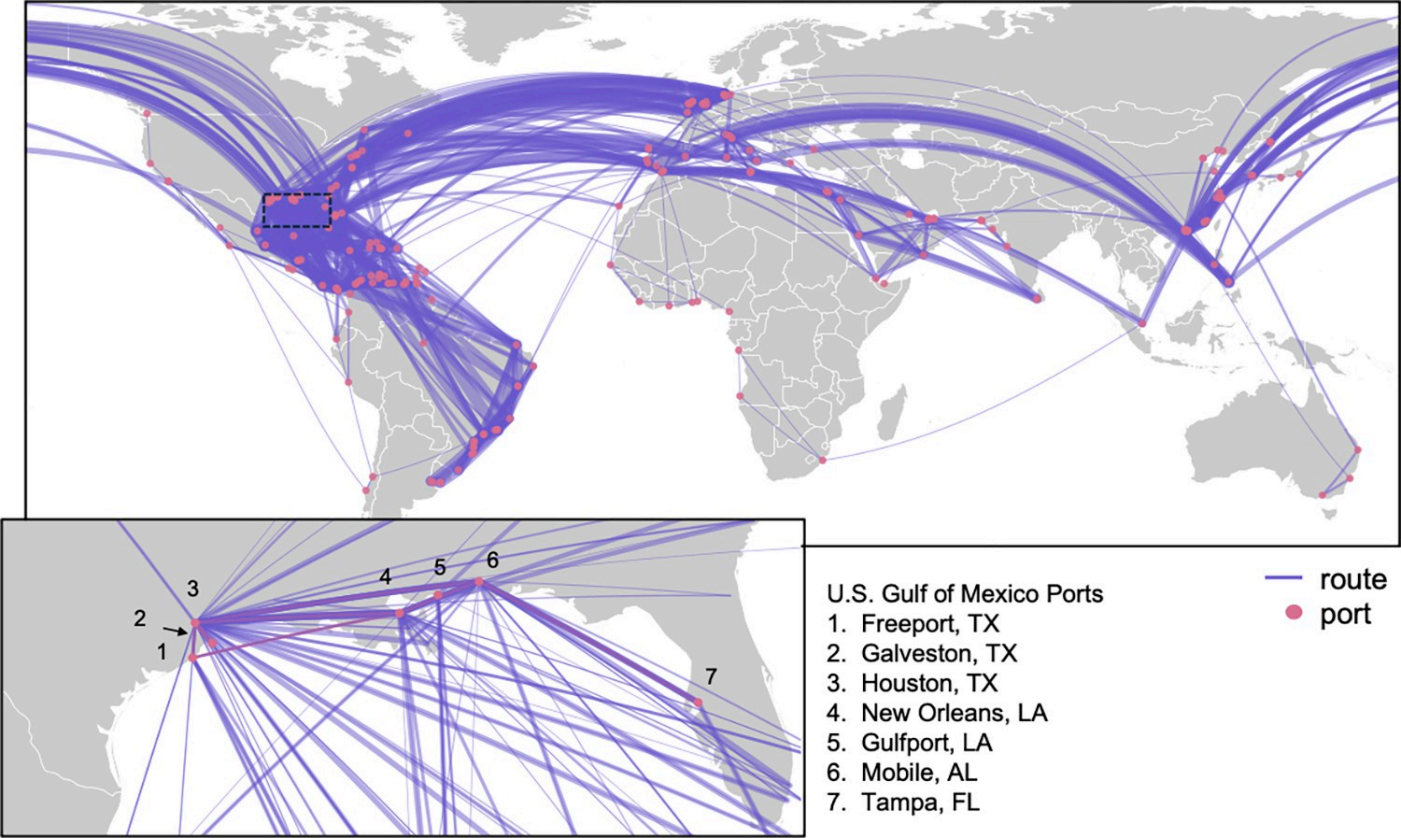
The Global Shipping Network containing Gulf State ports included 213 ports located in 69 countries. Lines are logarithmically weighted to demonstrate connectivity between ports and indicate a highly connected shipping network. Inset map shows the U.S. Gulf of Mexico ports and is highlighted by the dotted-line box in the large map. Map created in the R package ‘maps’ [42] using basemap data from Natural Earth (www.naturalearthdata.com).

The Gulf Coast of the United States (Fig 1) has been identified as a region at risk for the emergence and establishment of Zika virus and other arboviruses associated with *Aedes* spp. mosquitoes due to its warm and humid climate as well as the presence of many key transportation hubs (airports and seaports) within the region [31,32]. Although there have been outbreaks of Zika, dengue, and chikungunya in the U.S. Gulf States, these outbreaks do not compare in magnitude with those experienced in nearby Latin America [33–36]. This discrepancy may be partially explained by varying vector control and public health efforts as well as differences in housing style and lifestyle between affected countries, but is likely also partly due to differences in vector competence between mosquito populations [37–40]. Variation in vector competence between populations can occur at relatively fine spatial scales [41], meaning that continuous reintroduction of vectors into a region can influence local mosquito competency for various viruses. Thus, halting the genetic flow between disparate mosquito populations will aid in preventing the establishment of arboviral diseases in new locations. Developing models that predict reinvasions by *Ae*. *aegypti* and *Ae*. *albopictus* and identify the best strategies for targeted biosurveillance and vector control in ports could help to alert public health officials to potential threats and support optimized biosecurity efforts to halt reinvasion.

The goal of this study was to first integrate available *Aedes* species distribution data and maritime movement data to identify ports at high risk for importation of *Ae*. *albopictus* and *Ae*. *aegypti* via the GSN. Following this, we sought to understand how invasion risk could be minimized at high-risk ports and ports connected to these high-risk areas using biosurveillance. To do this, we used an agent-based model to quantify how port detection and removal protocols could be used to limit the establishment of mosquito vectors in new areas. This work may help officials concentrate biosecurity efforts to prevent further mosquito invasion and potential importation of vector-borne pathogens.

## Methods

We obtained data from Informa (formerly Lloyd’s Maritime Intelligence Unit; Informa, London, UK) detailing every fully cellular container ship that arrived at a major US port on the Gulf of Mexico in 2012 using automatic identification system transponders. We used these data and pathway-based, first-order Markov models to determine which ports along the US Gulf Coast were at the highest risk for importation of *Ae*. *aegypti* and *Ae*. *albopictus* along with container cargo shipped via maritime trade routes during 2012. Given that a ship loads and unloads cargo with each stop, our models also assume that some potential exists for infestation of the ship by mosquitoes at each stop at a port occupied by these species. These models therefore assume that some transmission potential exists between each port occupied by these species, and all ports visited subsequently. Thus, given a route A-B-C-D, where point D is the final port of call in the Gulf of Mexico and B is a port where at least one species of mosquito is present, we assume some potential for transmission from B to C and then from C to D. Because there is also some probability of cargo containing the mosquitoes to be unloaded at each port, we considered all points on a route together, running from *i* to *j*. This information was then used to assemble a database of routes *i* to *j* and the number of trips made by vessels along these routes.

Each route, *i* to *j*, had an associated number of stops *ij*. Each port occupied by either *Ae*. *aegypti* or *Ae*. *albopictus* was assigned constant transmission potential (*λ*) which was used to calculate the potential for importation (*P*_*ij*_) of each *Ae*. *aegypti* and *Ae*. *albopictus* into each one of our seven target ports. We then estimated the total relative likelihood of arrival by each species into each target port (*φ*_*j*_) by summing *P*_*ij*_ for all trips into each target port. Finally, we evaluated our model parameterization by generating multiple values for *λ*, and then generating a correlation matrix for *φ*_*ij*_ using Spearman rank correlation coefficients; models were robust to changes in parameterization (*r*_*s*_ > 0.964).

We also designed a simple agent-based model to explore how changes in probability of detecting and eradicating mosquitos on container ships influenced new mosquito population establishment. In our model, shipping containers aboard maritime ships were treated as agents, and mosquitoes found in containers on maritime ships were potentially moved among ports. Each container started its journey with an undefined number of mosquitos that was modeled with the assumption that the number of mosquitos was sufficient to support establishment at new ports. These containers were then moved between ports, and simulated mosquito detection, removal, and establishment procedures were modeled, and outcomes summarized across replicates.

For each iteration of the model, we moved ships and their containers between 1 to 10 ports to focus on control and establishment probabilities for mosquitos within the U.S. Gulf of Mexico ports (Table 1). Between each port of call, the mosquito population inside each container had a ~90% chance of surviving at sufficient numbers to support establishing a new land-side population; survival probability of mosquitos in each container was determined as a random deviate from a normal distribution with an initial mean of 0.9 and standard deviation of 0.035. For each container, the mean of the normal distribution was modified by adjusting a randomly assigned trip length, and this was meant to mimic decreased survival probability over for trips that took longer compared to trips that were quicker. Trip distance was randomly selected from our empirical distribution of trip distances, and this distance value was scaled by dividing it by the maximum trip length*10. The resulting survival penalty was then subtracted from the initial mean of 0.9 to calculate the final survival probability estimate for each container. These penalties had the potential to range from 0 to 0.1, with a mean survival probability penalty due to trip length of 0.012. In our model, the final modified survival probability was bounded by 0 and 1. The overall survival probability was based on an observed 85% survival rate using the optimal method for mailing mosquitos for research or management, but was set slightly higher to be more conservative in our model outputs [43]. Because of the uncertainty of the survival probability for mosquito populations in cargo containers specifically, we also assessed the sensitivity of these parameter choices on population establishment probabilities.

Once a ship entered a port, each container on the ship was processed in one of two ways: containers remained on the ship or were unloaded into the port. Containers remaining on board, which constituted 50% of containers, remained in their current state until the next survival check. Containers moved to shore were subjected to inspections to facilitate a mosquito search and removal procedure. In practice, containers are not inspected immediately upon movement to shore, and so we included an additional survival check for unloaded containers that occurred prior to any mosquito detection and removal operations. This was meant to include the possibility that mosquito populations may not survive in the containers during the port-side wait time. For this survival check, survival probability of mosquitos in each container was determined as a random draw from a normal distribution with a base mean of 0.85 and standard deviation of 0.035, again based on the observed 85% survival rate for mailing mosquitos [43].

For containers with surviving mosquito populations, we instituted a detection/removal procedure. To understand how sensitive these processes need to be to be broadly effective at significantly reducing new introductions, we considered efforts that ranged from ineffective (0% probability of detecting and removing mosquitos when they are present) to perfectly effective (100% probability of detecting and removing mosquitos when they are present), at 20% intervals (S1 Table). Here, we do not define the specific detection or removal methods to allow flexibility in applying our model outputs to many different mosquito targets, environments, management goals, and port regulations. When mosquito-laden containers were moved to shore and detection/control efforts failed, these populations were given the chance to establish a land-side population. Mosquito population establishments were successful for approximately 90% of attempts, which we modeled as a random deviate from a normal distribution with a mean of 0.9 and standard deviation of 0.05. We chose a 90% establishment probability based on the suitability of environmental factors in the region and predicted range of *Aedes spp*. [18]. However, similar to the in-cargo survival uncertainty testing, we also assessed the influence of this parameter value on the overall pattern of population establishment.

We ran each unique set of parameter combinations 100 times to generate estimates of the probability of establishment or re-establishment of *Aedes* at each port. All of these simulations were conducted entirely in the program R version 4.3.1 using base R packages [44].

## Results

We analyzed data detailing every fully cellular container ship that arrived into major US ports in the Gulf of Mexico between January 1^st^ and December 31^st^, 2012. These data were recorded by automatic identification system transponders, which are installed on every large ship and at every port and canal in world and automatically report data on ship size, location. Our data included the previous ten ports of call for each ship before arriving in one of seven US ports (Fig 1). We documented 1,921 arrivals and departures of 204 container ships. Using *Aedes* habitat suitability maps [18], we determined that only 39 (18.3%) of the 213 ports within our network (distributed across 69 countries) were likely to be free of *Ae*. *aegypti* and *Ae*. *albopictus* populations; for ports within our network, 140 (65.7%) were located in habitats that support populations of *Ae*. *aegypti*, 148 (69.4%) were located in habitats that support populations of *Ae*. *albopictus*, and 114 (53.5%) were located in habitats that support populations of both *Ae*. *aegypti* and *Ae*. *albopictus* (S1 Fig).

### Invasion risk assessment

We used pathway-based, first-order Markov models to determine which ports along the US Gulf Coast were at the highest risk for importation of *Ae*. *aegypti* and *Ae*. *albopictus* along with container cargo shipped via maritime trade routes during the year examined, 2012. Given that a ship loads and unloads cargo with each stop, our models also assume that some potential exists for infestation of the ship by mosquitoes at each stop at a port occupied by these species; because there is also some probability of cargo containing the mosquitoes to be unloaded at each port, we considered all points on a route together to fully understand invasion patterns. We determined that port traffic is a strong indicator of probability of invasion; we found a strong correlation between the total number of cargo ship arrivals at each port and likelihood of arrival by *Ae*. *aegypti* (r^2^ = 0.999 P > 0.0001) and *Ae*. *albopictus* (r^2^ = 0.999, P > 0.0001) (Table 1) Of the 1,921 arrivals into target ports, 99.2% of ships (n = 1,905) were moving from ports occupied by both *Ae*. *aegypti* and *Ae*. *albopictus* populations and only one arrival was coming from a port where neither species is commonly found (Table 2). Combined, these data suggest high probability of invasion potential, including movement of mosquitoes from a previously invaded location to a new or other previously invaded location.

**Table 1:** Ports along the Gulf Coast of the US with the highest relative likelihood of arrival (*φ*_*j*_) by *Ae*. *aegypti* and *Ae*. *albopictus* via the international maritime trade network given a constant transmission potential (*λ*) of 0.5. The total number of arrivals of fully cellular container ships at each port was strongly correlated with relative likelihood of arrival by both *Ae*. *aegypti* (r^2^ = 0.999, P > 0.0001) and *Ae*. *albopictus* (r^2^ = 0.999, P > 0.0001) during this time frame. This was reflective of the high connectivity between ports, which implies high risk for movement of *Aedes* spp. mosquitoes between these cities. While Houston seems to play a role as a hub for international arrivals, New Orleans and Mobile receive a great number of shipments from domestic ports, including Houston. Count data represents arrivals by fully cellular container ships from January 1^st^ to December 31^st^, 2012.

U.S. Gulf of Mexico Port	φ_j_ (*Aedes aegypti*)	φ_j_ (*Aedes albopictus*)	Total arrivals	Number of trips from U.S. Gulf of Mexico Port:						
				Houston, TX	New Orleans, LA	Mobile, AL	Gulfport, MS	Freeport, TX	Tampa, FL	Galveston, TX
Houston, TX	885.75	909.39	985	--	316	98	0	6	0	2
New Orleans, LA	422.34	420.3	435	89	--	50	0	0	0	0
Mobile, AL	236.73	240.07	252	32	39	--	0	0	7	0
Gulfport, MS	132.52	135.54	136	0	0	0	--	0	0	0
Freeport, TX	54.95	54.52	55	0	6	0	0	--	0	0
Tampa, FL	55.93	50.51	50	0	0	48	0	0	--	0
Galveston, TX	1.99	1.85	2	2	0	0	0	0	0	--

### Invasion risk mitigation

We designed an agent-based model to understand how to effectively mitigate mosquito invasion risk. In the model, shipping containers aboard maritime ships were treated as agents, where mosquitoes found in these vessels could be moved between ports. Each container was assumed to start its journey with enough mosquitoes that mosquito establishment at new ports was theoretically possible. Containers moved between ports and could be moved to shore at any port with varying probabilities.

**Fig 2:**
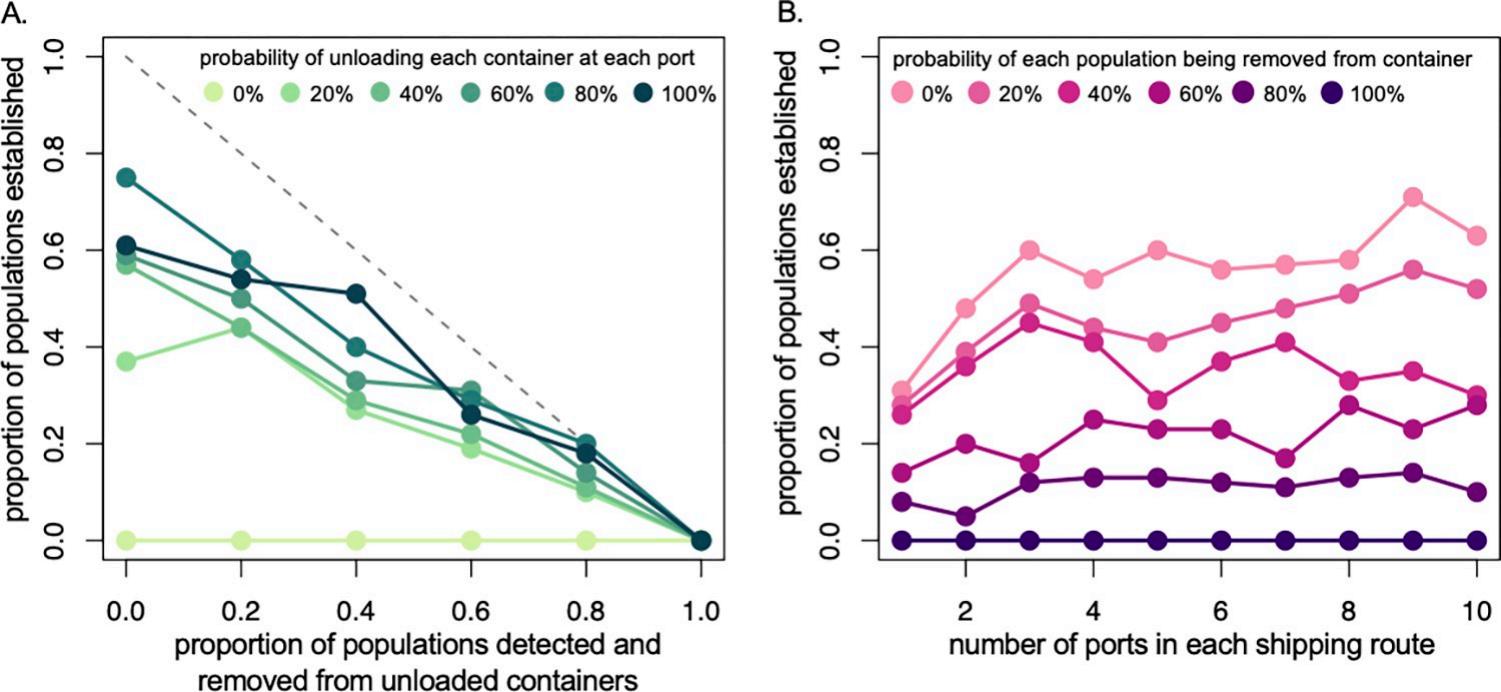
Effectiveness mosquito detection and removal programs displayed as the proportion of populations that were established from containers that initially contained viable mosquito populations. A) As the effectiveness of mosquito detection and removal increased, the proportion of mosquito populations decreased even when the probability of a container being move to shore was as low as 20%. B) The number of stops at ports on a shipping route generally did not interact with the probability of mosquito population detection and removal to influence mosquito population establishment rate. However, when detection rates were low and there were relatively few stops, the number of stops was positively related to the proportion of mosquito populations established.

To combat the establishment of new mosquito vector populations at these simulated ports, we enacted port procedures for detecting and removing mosquitoes from shipping containers. Containers were checked upon arrival to shore, with a probability of removing mosquitoes ranging from 0 to 100% for each event. We found that detection and removal of mosquito infestations after unloading at the destination reduced the probability of mosquito establishment (Fig 2A). The overall pattern of these trends was not sensitive to the initial survival probability of mosquitos in containers or of survival of populations on the shore, although decreased survival generally dampened the observed trends (S2 and S3 Figs). Thus, onshore mosquito detection and control efforts in maritime goods is capable of preventing new introductions of mosquito populations at shipping ports.

We also compared the number of ports a ship visited to mosquito population establishment probability. In our model and in reality, the number of stops a ship makes controls the number of opportunities for transfer between ships and land and, therefore, the opportunities for mosquito detection, removal, and establishment. We found that the number of stops generally did not interact with the effect of removing mosquitoes from unloaded containers and that population establishment increased with decreasing removal probability (Fig 2B). As before, the overall pattern of these trends was not sensitive to the initial survival probability of mosquitos in containers or of survival of populations on the shore (S2 and S3 Figs). However, when detection rates were <40% and there were relatively few stops (<4), the number of stops was approximately linearly and positively related to the proportion of mosquito populations established. Overall, this suggests that improvements in detection and removal effectiveness will decrease introductions, even as global shipping networks continue to grow and incorporate new ports and routes, and that increasing detection and removal effectiveness for the shortest routes may has the potential to decrease introduction risks the fastest.

**Table 2:** Ports with the highest immediate connectivity to our seven target ports in the US Gulf States. Since nearly all maritime arrivals in the Gulf passed most recently through ports on the Atlantic seaboard, in the Caribbean, or in other ports on the Gulf of Mexico, all of which host populations of both *Ae*. *aegypti* and *Ae*. *albopictus*, mosquito populations from these ports must reasonably be assumed to be the most likely to arrive in target ports. Data represents arrivals by fully cellular container ships from January 1^st^ to December 31^st^, 2012.

International Ports or U.S. Ports Outside Gulf of Mexico	Total trips to target ports	Trips to Houston	Trips to New Orleans	Trips to Mobile	Trips to Gulfport	Trips to Tampa
Altamira, Mexico	373	364	0	9	0	0
Santo Tomás de Castilla, Guatemala	160	130	29	0	1	0
Puerto Cortes, Honduras	105	3	5	0	96	0
Savannah, Georgia, USA	104	102	0	2	0	0
Kingston, Jamaica	102	17	18	18	0	49

## Discussion

In our models, connectivity as measured by frequency of ship arrivals and previous ports of call, predicted likelihood of mosquito invasion and this has important ramifications for eventual invasion by *Aedes* species. For example, the Port of Houston, Texas represented by far the greatest risk for the invasion of *Ae*. *aegypti* and *Ae*. *albopictus* to other US ports along the coast of the Gulf of Mexico. In our 2012 dataset, the Port of Houston received more than double the arrivals of container ships than the U.S. Gulf port with the next most arrivals, New Orleans. In fact, Houston received more arrivals during this period than all six other major U.S. ports in the Gulf combined (Table 1). While more than three-quarters of container ships arriving in Houston had most recently come from a port outside the Gulf which hosted both *Ae*. *aegypti* and *Ae*. *albopictus*, the majority of traffic into other Gulf ports was internal, with arrivals coming from other Gulf ports (Tables 1 and 2). These data are in line with other historical data on frequency of container ship arrivals and cargo tonnage, which show that Houston received more arrivals and handled more tonnage than any other port in the Gulf from 2016–2018, and that Houston handled a higher proportion of foreign arrivals and freight than did other Gulf ports [45].

While the total number of arrivals by container ships may not always indicate the highest likelihood of arrival by invasive species generally, the distributions and common occurrence of *Ae*. *aegypti* and *Ae*. *albopictus* within our network (S1 Fig) led to a high correlation between these variables. Thus, ports with the highest connectivity to our target ports along the U.S. Gulf Coast are likely to play a disproportionate role in the dispersal of invasive mosquitoes to our target ports. Because the probability of unloading infested cargo from a given port diminishes with each unloading visit along a cargo ship’s route, and because accompanying invasive mosquitoes are most likely to survive and disperse given shorter travel times [22], we assumed (and modeled because first order Markov models are inherently weighted by distance) that ports most immediately visited by ships prior to arrival in US Gulf ports pose the greatest relative risk for importation of *Ae*. *aegypti* and *Ae*. *albopictus*.

While most ports on the US Gulf Coast have relatively little immediate connection to ports outside the Gulf Coast region, the high level of connectivity among several US Gulf ports (Tables 1 and 2) may provide a vehicle for dispersal of invasive species into ports with less outside connectivity. Because *Ae*. *aegypti* and *Ae*. *albopictus* are so widely distributed among port cities, and especially those connected to ports in the US Gulf Coast, implementation of origin-specific screening is unlikely to lead to increased efficiency in halting the dispersal of these species into the US Gulf Coast region. Instead, preventing mosquitoes from entering the US Gulf Coast network seems particularly critical. Since Houston serves as a hub for vessels entering the US Gulf Coast network, implementation of an early alert and rapid response system for screening ships entering the Port of Houston could disproportionately reduce the risk of maritime dispersal of invasive species, including *Ae*. *aegypti* and *Ae*. *albopictus*. The findings from the agent-based model support the screening vessels upon arrival as a strong intervention to reduce establishment of mosquito populations. Combined with the Markov model findings, mosquito removal of container cargo upon arrival to the Port of Houston could serve as an effective strategy at reducing invasive mosquito populations in the US Gulf Coast.

Our analysis underscores the critical role of global shipping networks, particularly through hubs like the Port of Houston, in the potential dispersal of invasive mosquito species such as *Ae*. *aegypti* and *Ae*. *albopictus*. This connectivity not only enhances the risk of invasion by these vectors but also serves as a conduit for the diseases they carry, notably dengue and chikungunya [46]. Fredericks and Fernandez-Sesma [44] argued for increased vigilance at ports that serve as entry points for these vectors into new regions as a method for reducing the spread of arboviruses. This aligns with our suggestion for implementing an early alert and rapid response system for ships entering the Port of Houston, as this could serve as a critical preventative measure against the dispersal of *Ae*. *aegypti* and *Ae*. *albopictus*. Furthermore, historic outbreaks of dengue in Texas [47,48] and the autochthonous transmissions that have occurred in Texas in the last 15 years [49] support our findings that the highly connected nature of the Port of Houston warrants increased vigilance to combat the spread of vector-borne diseases. The interconnectedness of global shipping networks in combination with the epidemiology of diseases such as dengue and chikungunya underscores the importance of integrating port-based biosecurity measures with broader public health strategies aimed at vector control and disease prevention [46].

While our results accurately reflect the movements of all fully cellular container ships that arrived in the seven target ports along the US Gulf Coast, a number of potential routes of dispersal and potential vectors for dispersal were not considered in our study. First, our data did not include information on the movements of non-containerized cargo along the GSN. However, container ships are often considered to be better than non-containerized cargo ship as vectors for the dispersal of terrestrial invasive species because containers are rarely, if ever, opened and examined between destinations [50]. In addition, it is likely that our agent-based model conclusions, namely that screening at final destinations for mosquito infestations are the best way to prevent new invasions, holds true for smaller ships as these ships also visit ports within the *Aedes* spp. ranges.

Our models contained several assumptions and generalizations necessitated by data availability and the general lack of knowledge regarding transport of mosquitoes in cargo. Specifically, data collected by an automatic identification system does not include information on the number of containers or the type of cargo carried by each ship, so we assumed that each ship had the same capacity for infestation and transmission. Furthermore, these records do not include information on whether cargo was loaded or unloaded at each port. Some port visits are made for purposes of refueling, and involve no transfer of cargo to or from the vessel [51]. In addition, transmission potential is likely to vary with environmental conditions, and this was not included in our model. As a result of these limitations to our data, we assumed a constant probability for transmission from one port where a mosquito occurred to the next port, while in reality this probability is certainly heterogenous. This assumption was also present in our agent-based model, as was the feasibility of mosquito removal and screening of containerized cargo. Finally, the inherent complexities of modeling biological and environmental systems means that that model validation is an ongoing process, and here we emphasize that no model, including ours, can perfectly predict real-world outcomes. This understanding underscores the importance of continuous refinement and validation of our models, particularly as new data and insights become available, to improve their accuracy and relevance to public health strategies against vector-borne diseases. More detailed information on containers and cargo, as well as quantification of mosquito infestation of these cargo, would dramatically improve our model and provide more insight into paths utilized by *Ae*. *aegypti* and *Ae*. *albopictus* for dispersal.

## Conclusions

This study represents the first pathway-based analysis of dispersal by *Ae*. *aegypti* and *Ae*. *albopictus* into and among major ports on the US Gulf Coast via the global shipping network. These mosquitoes, which are the primary vectors of numerous arboviruses that affect human an animal health [13–15], are also some of the most invasive insects on earth [9,19]. Understanding long-distance dispersal of these species via maritime trade allows us to concentrate biosecurity and vector control efforts, thereby increasing management efficiency, and may allow us to better understand gene flow and patterns of population genetics and phenotypic traits that are important for mosquito control and public health [52]. For example, understanding how traits that convey resistance to insect control methods evolve and are spread between populations and regions is critical to the long-term effectiveness of mosquito control programs. We also show that port-based detection and control of potential mosquito invaders can substantially reduce the risks of martime-based invasion. A number of highly invasive and medically important mosquitoes, including *Anopheles stephensi* (Liston), *Ae*. (Hulecoeteomyia) *koreicus* and *Ae*. (Finlaya) *japonicus japonicus* (Theobald), are currently expanding their global ranges both over land and through long-distance dispersal via the GSN [53–55]. By understanding vector dispersal and its downstream effects, we may better understand and prevent outbreaks of vector-borne pathogens.

## Supporting information

S1 Table Description of parameters and the values of each parameter we tested in our agent-based models. All pairwise combinations of values (in total 784,080 sets) were tested and replicated 100 times. Default parameters values were used when isolating the effects of individual parameters on mosquito population establishment.
(DOCX)

S2 Table Maritime transport data from ship transponders and shore receivers used in these analyses. Columns include an individual ship identification number, a column noting the arrival port and a column noting the departure port.
(CSV)

S1 Fig Using predicted *Aedes* distributions maps [18], we determined that only 39 (18.3%) of the 213 ports within our network (distributed across 69 countries) were likely to be free of *Ae*. *aegypti* and *Ae*.*albopictus* populations; 140 (65.7%) ports within our network had suitable habitats for *Ae*. *aegypti*, 148 (69.4%) had suitable habitats for *Ae*. *albopictus*, and 114 (53.5%) ports had suitable habitats for both *Ae*. *aegypti* and *Ae*. *albopictus*. Map created in the R package ‘maps’ [42] using basemap data from Natural Earth (www.naturalearthdata.com).
(TIFF)

S2 Fig Analysis of the initial mosquito survival probability in cargo containers value on patterns of mosquito population establishment. Initial survival probability for each row of panels is noted on the left side. A, C, E, G, I) Effects of the proportion of mosquitos detected and removed from unloaded cargo on the rate of mosquito population establishment. B, D, F, H, J) Effect of the number of stops at ports on a shipping route on mosquito population establishment rate. Initial survival probability influenced the magnitude of the patterns observed in population establishment, the qualitative relationship between parameters and values remained the same among these comparisons.
(TIFF)

S3 Fig Analysis of the effects of survival probability of a mosquito population that made it to shore becoming on patterns of mosquito population establishment. The value of this parameter is noted on the left side of each row of panels. A, C, E, G) Effects of the proportion of mosquitos detected and removed from unloaded cargo on the rate of mosquito population establishment. B, D, F, H) Effect of the number of stops at ports on a shipping route on mosquito population establishment rate. On-shore survival probability slightly influenced the magnitude of the patterns observed in population establishment, but the qualitative relationship between parameters and values remained the same among these comparisons.
(TIFF)
