# Supplementary material for: Assessing and managing the risk of Aedes mosquito introductions via the global maritime trade network
Source: PLoS Negl Trop Dis. 2024 Apr 10;18(4):e0012110. doi: 10.1371/journal.pntd.0012110 (PMC11034661; doi:10.1371/journal.pntd.0012110)
Supplement: S1 Table — All pairwise combinations of values (in total 784,080 sets) were tested and replicated 100 times. Default parameters values were used when isolating the effects of individual parameters on mosquito population establishment. (DOCX) [file pntd.0012110.s001.docx]

**Supplementary Materials 2.** Description of parameters and the values of each parameter we tested in our agent-based models. All pairwise combinations of values (in total 784,080 sets) were tested and replicated 100 times. Default parameters values were used when isolating the effects of individual parameters on mosquito population establishment.

| **parameter** | **value range** | **increment** | **default** |
| --- | --- | --- | --- |
| probability that a container was moved from ship to shore | 0-100% | 0.1 | 50% |
| probability of mosquitoes leaving an onshore container and establishing a viable population | 0-100% | 0.1 | 90% |
| number of stops on a ship route | 1-10 | 1 | 10 |
| probability of mosquitoes surviving the journey between ports | 0-100% | 0.1 | 90% |
| probability of detecting and removing mosquitoes from on-land container | 0-100% | 0.1 | 90% |
