# Supplementary figures and images for: Assessing and managing the risk of Aedes mosquito introductions via the global maritime trade network
Source: PLoS Negl Trop Dis. 2024 Apr 10;18(4):e0012110. doi: 10.1371/journal.pntd.0012110 (PMC11034661; doi:10.1371/journal.pntd.0012110)

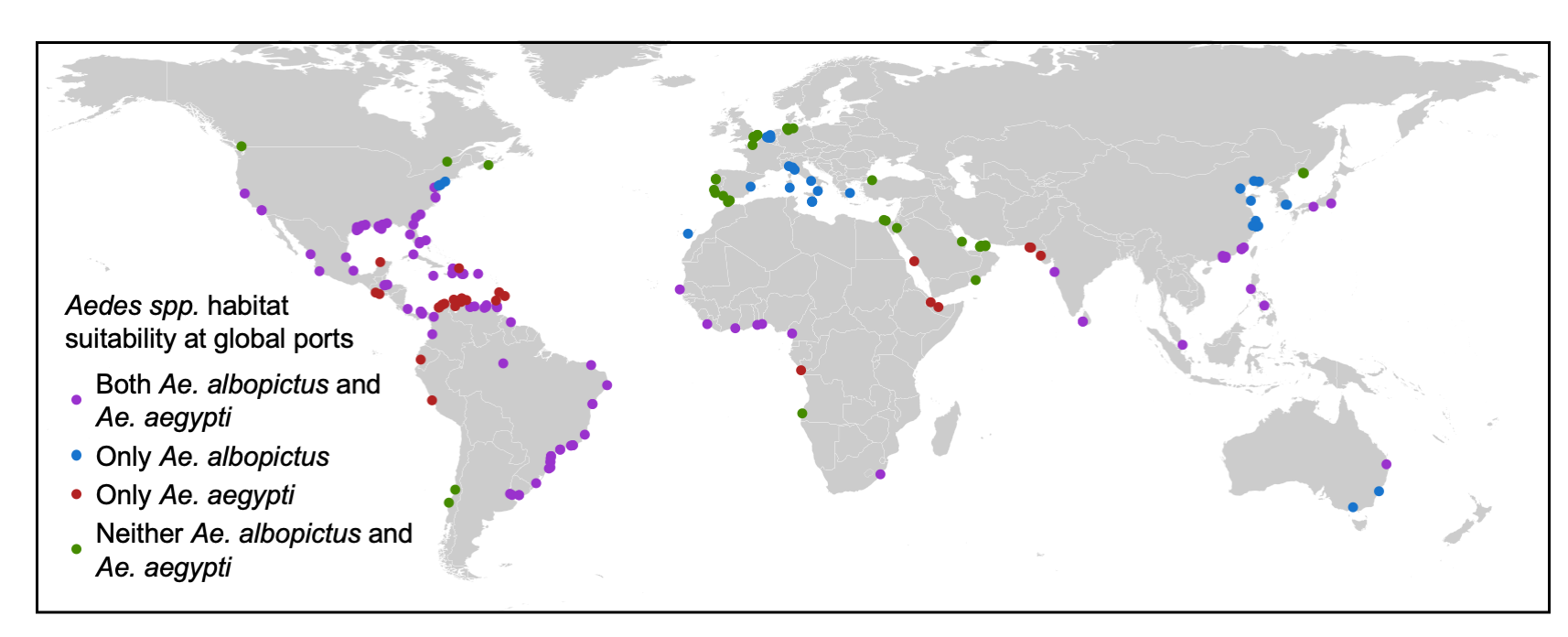

Supplement: S1 Fig — Using predicted Aedes distributions maps [18], we determined that only 39 (18.3%) of the 213 ports within our network (distributed across 69 countries) were likely to be free of Ae. aegypti and Ae.albopictus populations; 140 (65.7%) ports within our network had suitable habitats for Ae. aegypti, 148 (69.4%) had suitable habitats for Ae. albopictus, and 114 (53.5%) ports had suitable habitats for both Ae. aegypti and Ae. albopictus. Map created in the R package ‘maps’ [42] using basemap data from Natural Earth (www.naturalearthdata.com). (TIFF) [file pntd.0012110.s003.tiff]

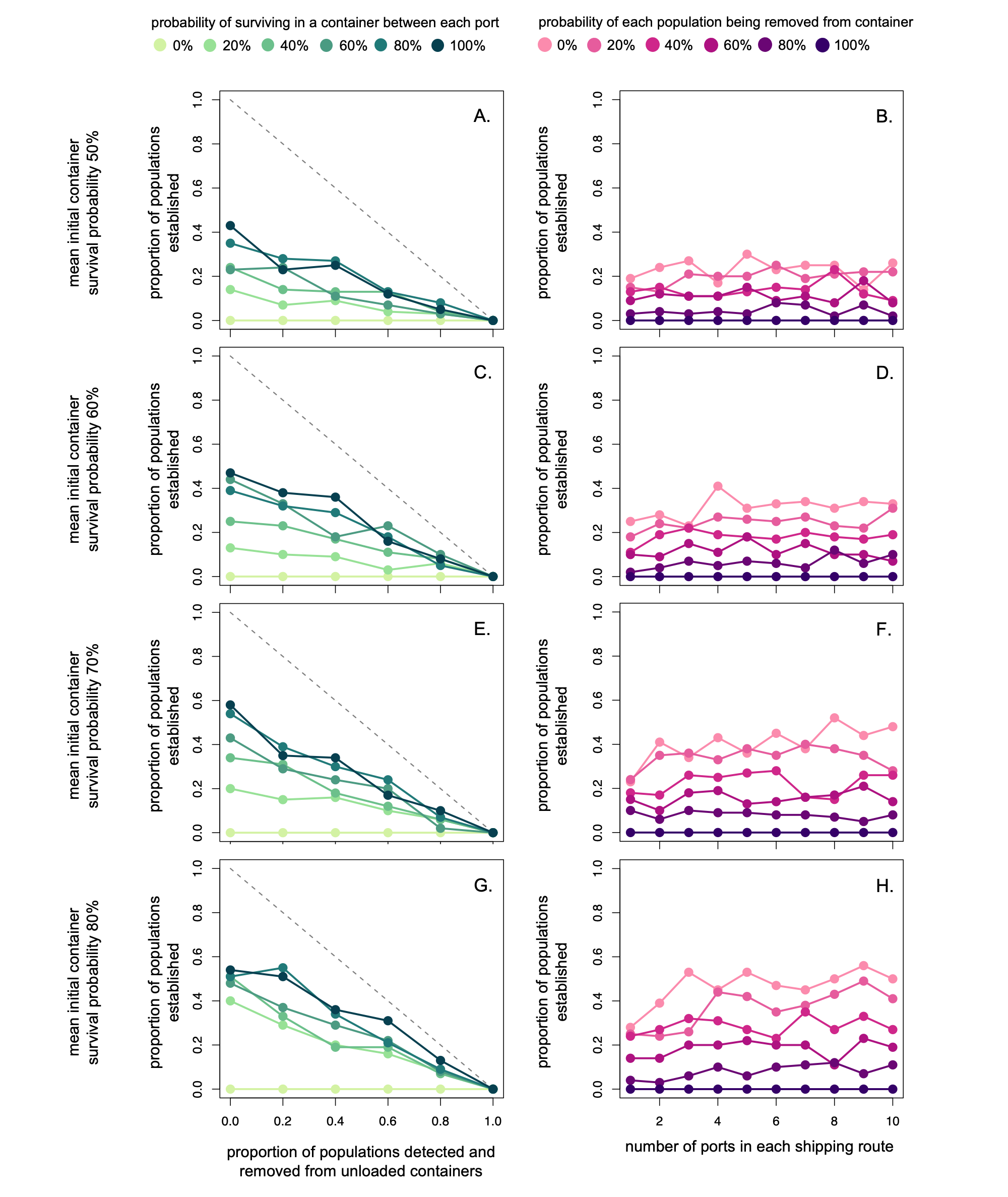

Supplement: S2 Fig — Initial survival probability for each row of panels is noted on the left side. A, C, E, G, I) Effects of the proportion of mosquitos detected and removed from unloaded cargo on the rate of mosquito population establishment. B, D, F, H, J) Effect of the number of stops at ports on a shipping route on mosquito population establishment rate. Initial survival probability influenced the magnitude of the patterns observed in population establishment, the qualitative relationship between parameters and values remained the same among these comparisons. (TIFF) [file pntd.0012110.s004.tiff]

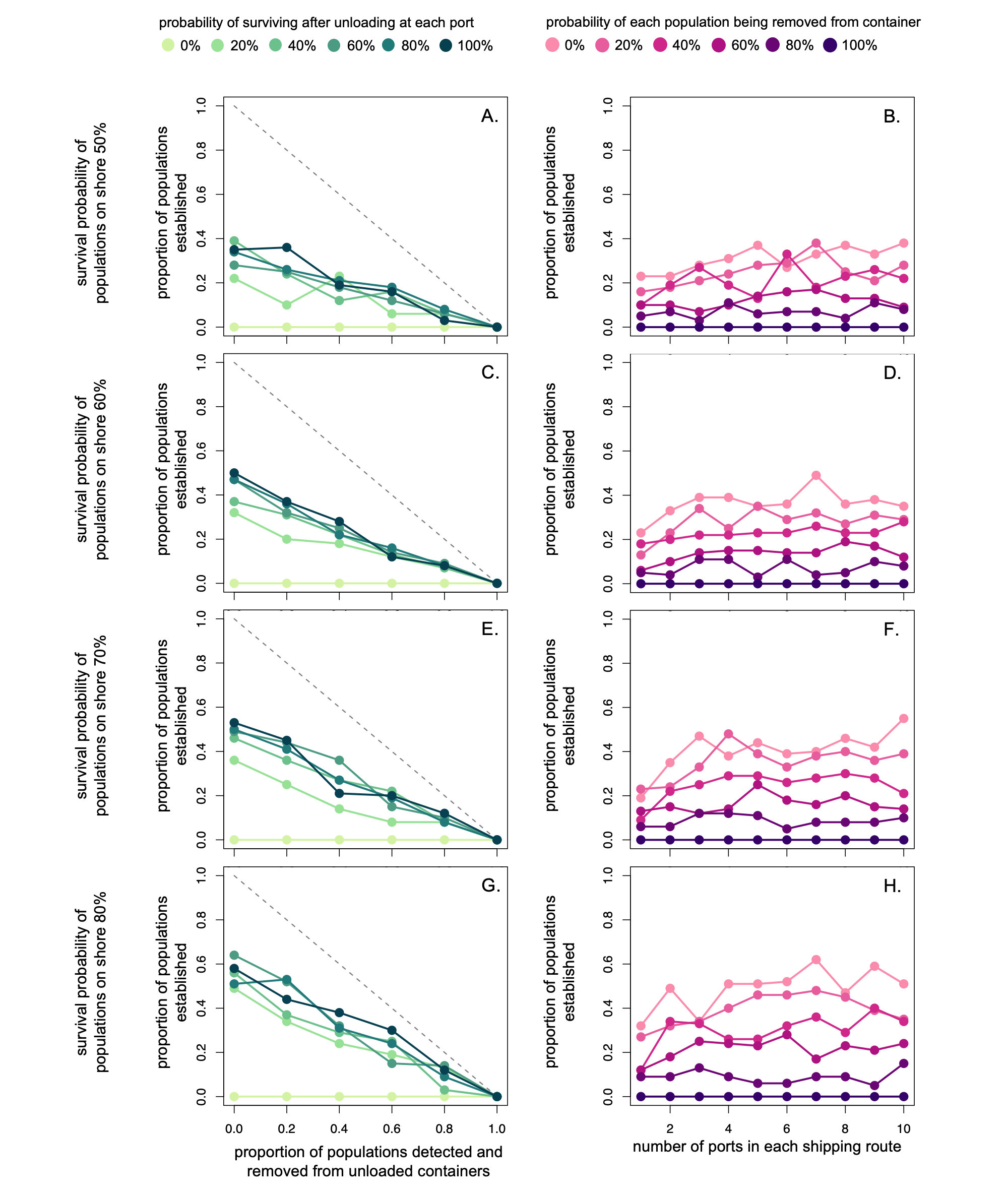

Supplement: S3 Fig — The value of this parameter is noted on the left side of each row of panels. A, C, E, G) Effects of the proportion of mosquitos detected and removed from unloaded cargo on the rate of mosquito population establishment. B, D, F, H) Effect of the number of stops at ports on a shipping route on mosquito population establishment rate. On-shore survival probability slightly influenced the magnitude of the patterns observed in population establishment, but the qualitative relationship between parameters and values remained the same among these comparisons. (TIFF) [file pntd.0012110.s005.tiff]
